# Books and Pamphlets Received

**Published:** 1893-04

**Authors:** 


### BOOKS AND PAMPHLETS RECEIVED.

"Bloodless Amputation at the Hip Joint by a New Method," by N. Senn, M. D., Chicago, Ill·

"Modern Homeopathy; its Absurdities and Inconsistencies," by W. W. Browning, M. D., Brooklyn, N. Y.

"Irrigation of Urethra and Bladder by Position and Continuous Current," by H. G. Daggett, M. D., Buffalo N. Y.

"Pshychopatphia Sextualis," by Krafft-Ebing.

"An American Text Book of the Practice of Medicine, Vol. I," by William Pepper, M. D., Philadelphia.

"The Year-Book of Treatment, 1893."

"Diseases of the Skin," by C. C. Ransom, M. D., New York.

"Elementary Physiology," by A. T. Schofield, M. D., M. R. C. S. etc.

"Life of D. Hayes Agnew," by J. Howe Adams, M. D., Phil adelphia.

"Senile Hypertrophied Prostate," by Bransford Lewis, M. D., St. Louis, Mo.

"Introductory Address at the College of Physicians and Surgeons," Session 1892-3, by Prof. G. Frank Lydston, M. D., Chicago, Ill.

"The Third Year's Work at the Clinic for Diseases of the Rectum in the New York Post-Graduate Hospital," by Charles B. Kelsey, M. D., New York.

"The Bicycle and its Relation to the Physician," by Seneca Egbert, A. M., M. D., Philadelphia.

"An Inquiry into the Relative Merits of Vaginal Hysterectomy and high Amputation or Partial Extirpation by Galvano-Cautery in Cancer of the Cervix Uteri," by John Byrne, M. D., M. R. C. S., Brooklyn, N. Y.

